# Antibacterial Effect of Iodine‐Based Paste With Silver Nanoparticles Against *Enterococcus faecalis*: An In Vitro Study

**DOI:** 10.1002/cre2.70410

**Published:** 2026-07-16

**Authors:** Carbajal‐Bastida Sarasuadi, Robles‐Bermeo Norma Leticia, Guadarrama‐Reyes Saraí Carmina, Morales‐Luckie Raúl Alberto, María Guadalupe González‐Pedroza, Bermeo‐Escalona Josué Roberto, Carlo Eduardo Medina Solís

**Affiliations:** ^1^ Master's Program in Odontological Sciences, Faculty of Dentistry, Keisaburo Miyata Center for Research and Advanced Studies in Odontology Autonomous University of the State of Mexico Toluca Mexico; ^2^ Faculty of Dentistry, Keisaburo Miyata Center for Research and Advanced Studies in Odontology Autonomous University of the State of Mexico Toluca Mexico; ^3^ Faculty of Dentistry Autonomous University of the State of Mexico Toluca Mexico; ^4^ Department of Dental Materials Science, Center for Research in Sustainable Chemistry (CCIQS) Autonomous University of the State of Mexico (UAEMex) Toluca Mexico; ^5^ Department of Biotechnology, Faculty of Sciences Autonomous University of the State of Mexico (UAEMex) Toluca Mexico; ^6^ Academic Unit of Dentistry, Institute of Health Sciences Autonomous University of the State of Hidalgo Pachuca de Soto Mexico

**Keywords:** antibacterial, *Enterococcus faecalis*, iodine‐based paste, silver nanoparticles

## Abstract

**Background:**

Pulpectomy is one of the most common treatments for the rehabilitation and conservation of primary dentition. This procedure requires thorough disinfection of the root canals to eliminate the bacterial load prior to obturation. However, the anatomical complexity of primary root canals limits the complete eradication of certain bacteria, such as *Enterococcus faecalis* (*E. faecalis*). Although iodine‐based pastes, such as Vitapex, are considered the gold standard for primary root canal fillings due to their excellent clinical outcomes, the extent of their antibacterial efficacy remains unclear.

**Objective:**

To evaluate the antibacterial efficacy of an iodine‐based paste incorporated with silver nanoparticles against *E. faecalis*.

**Design:**

This experimental study employed a comparative, cross‐sectional, analytical, and prospective design.

**Methods:**

Silver nanoparticles (AgNPs) were synthesized and chemically characterized prior to incorporation into an iodoform paste (Vitapex). The antibacterial efficacy was subsequently evaluated across three distinct groups: Group 1 (Vitapex), Group 2 (AgNPs), and Group 3 (Vitapex with AgNPs). Experiments were performed in triplicate using the *E. faecalis* ATCC 29212 strain to measure the zones of inhibition for each group.

**Results:**

Significant statistical differences were observed (*p* = 0.003) in the inhibition zones against *E. faecalis*. The antibacterial activity of the **Vitapex+AgNPs** combination (SD 13.93 ± 0.83) was significantly higher than that of **Vitapex** (SD 10.26 ± 0.88) and **AgNPs** (SD 11.15 ± 2.40) alone.

**Conclusion:**

Our results demonstrate that both Vitapex and AgNPs exhibit antibacterial activity against *E. faecalis*; furthermore, their combined application shows a superior synergistic effect.

## 1 Introduction

The complex balance between human microbiota species determines whether a healthy (symbiotic) or a disease‐related (dysbiotic) state develops and persists in the oral cavity (Camp 1984; Kratunova and Silva 2018).

The dysbiosis produced in the caries process alters the integrity of the dental organ and extends to the root canals, causing pulp inflammation, necrosis, or periradicular lesions.

Under normal conditions, the dentin‐pulp complex is sterile and isolated by the enamel and cementum. However, when its integrity is broken, the complex is exposed to the oral environment and becomes colonized by resident bacteria via the biofilm (Chávez‐Andrade et al. 2019).

Most bacteria in dental caries are immobile, infiltrating the dentin through division, while hydrostatic pressures during mastication drive them into the tubules. Likewise, when the crown fractures or the enamel develops cracks, numerous dentin tubules become exposed to the oral environment, facilitating bacterial invasion. Regardless of how bacteria access the root canal, pulp tissue necrosis is essential to the establishment of primary infections (Chávez‐Andrade et al. 2019).

Root canals of primary teeth with necrotic pulp and periapical lesions contain large numbers of microorganisms, typically forming polymicrobial infections with both aerobic and anaerobic bacteria (Arweiler and Netuschil 2016).

Initially, anaerobes such as Streptococcus, *Enterococcus faecalis (E. faecalis)*, Lactobacillus, and Corynebacterium are most prevalent; however, over time, other anaerobic species—particularly gram‐negative bacilli—become more abundant. As the carious lesion progresses, the predominant microbiota shift to strict anaerobes, including Fusobacterium, Porphyromonas, Prevotella, Eubacterium, and Peptostreptococcus (Yin et al. 2020; Khan et al. 2019). Bacterial species such as *E. faecalis* are commonly found in previously treated teeth and are linked to root canal failures (Arweiler and Netuschil 2016).

*Enterococcus faecalis* can survive in nutrient‐poor environments, tolerate alkaline pH up to 11.5, withstand temperatures from 10° to 60°C, and endure high salinity. It is capable of invading dentin tubules and persisting there for extended periods. Additionally, it can resist intracanal medications and survive as a lone microorganism in root canals (Kilian et al. 2016; Van'T Hof et al. 2014; Cruz et al. 2017).

This bacteria has been identified as the primary cause of endodontic failure owing to its resistance to disinfectants, its ability to form biofilms, and its persistence in regions beyond the reach of chemo‐mechanical root canal debridement (Kilian et al. 2016; Van'T Hof et al. 2014; Cruz et al. 2017).

The goal of pulpectomy in primary teeth is to preserve them until natural exfoliation. Indications include irreversible pulpitis, infection, or necrosis (Kenneth and Hargreaves 2022). Canals are disinfected with antibacterial solutions and mechanically cleaned to eliminate bacterial load, after which they are filled with a biocompatible, resorbable material to restore tooth function (Kenneth and Hargreaves 2022; Nair 2014).

When performing pulpectomies in primary teeth, the filling material should be radiopaque, antibacterial, biocompatible with surrounding tissues and the successor tooth, and resorb along the root canal anatomy. It should also be easy to manipulate and adhere well to the canal walls (Pazelli et al. 2003).

Considered the gold standard for filling primary root canals, Vitapex is an iodine‐based paste with a viscous consistency (Wu et al. 2014; Najjar et al. 2019). It is composed primarily of 40.4% iodoform, 30.3% calcium hydroxide, and 22.4% silicone, with silicone oil serving as the vehicle to prevent hardening and facilitate handling. When extruded into furcation or apical areas, it is resorbed by macrophages within 1 to 2 weeks. Bone regeneration following its use has been documented clinically and histologically (Nurko et al. 2000); however, its antibacterial efficacy has been questioned (Trejo and Cuevas 2014; Nurko et al. 2000). Incorporating silver nanoparticles (AgNPs) into certain materials has been shown to improve their antibacterial efficacy, even at low proportions (Afkhami et al. 2015; Jandt and Watts 2020).

No information currently exists on the incorporation of AgNPs into Vitapex. Therefore, the aim of this study was to evaluate the antibacterial effect of Vitapex combined with AgNPs against *E. faecalis*.

## 2 Materials and Methods

AgNPs were synthesized and characterized using UV‐Vis spectroscopy and transmission electron microscopy (TEM) to confirm their formation and determine their size and shape. The AgNPs were then incorporated into Vitapex, and the functional groups involved in their incorporation were analyzed using Fourier‐transform infrared (FTIR) spectroscopy

The antibacterial activity of three groups was evaluated: Group 1, Vitapex; Group 2, AgNPs; and Group 3, Vitapex combined with AgNPs, using the *E. faecalis* ATCC 29212 strain. All experiments were performed in triplicate.

## 3 Synthesis of Silver Nanoparticles

To synthesize the AgNPs, an aqueous solution of 100 mL of AgNO_3_ at a concentration of 1 × 10^−3^ M was prepared. Separately, 100 mL of NaBH_4_ at a concentration of 2 × 10^−2^ M was prepared. The reducing and oxidizing agents were then mixed in a 1:1 volume ratio, producing a greenish color indicative of AgNP formation. Finally, the AgNPs were collected by centrifugation and filtered using Whatman No. 5 filter paper to obtain particles of nanometric size (Merga et al. 2007; Iravani et al. 2014).

## 4 Incorporation of Silver Nanoparticles Into Vitapex

A solution of the non‐ionic surfactant (Brij 35) was prepared by dissolving 1 g in 100 mL of distilled water, followed by heating with constant stirring for 30 min until homogeneity was achieved. The mixture was then left to stand for 1 h. The incorporation of Vitapex, surfactant (1%), and AgNPs in a 1:1:1 molar ratio was then initiated, using a watch glass and a spatula. The components were stirred for 10 min until a uniform blend was obtained (Vinarov et al. 2018; Sekhon 2013).

## 5 Characterization of the Silver Nanoparticles

### 5.1 Spectroscopy UV‐Vis

Analysis was conducted using a spectrophotometer (VE‐5100UV, VELAB) operated at a resolution of 1 nm and maintained at room temperature. Spectral data were then collected over the 300–800 nm range, and reaction kinetics were monitored until stable AgNPs were obtained.

### 5.2 Scanning Electron Microscopy With Energy Dispersive Spectroscopy (SEM‐EDS)

The final product was adhered to aluminum stubs using conductive tape, coated with carbón, and examined using a scanning electron microscope (JEOL, JSM‐6510LV at 20 kV, Tokyo, Japan) with secondary electrons at 100×, 500×, and 3000× magnifications. EDS analysis was subsequently performed.

### 5.3 Transmission Electron Microscopy (TEM)

Micrographs were obtained using a JEOL JEM‐2100 microscope (Tokyo, Japan). The sample was prepared by placing a drop of the suspension onto a copper grid (300 mesh) coated with a carbon film and allowing it to dry at room temperature.

### 5.4 FTIR Analysis

FTIR spectra of the AgNPs incorporated into Vitapex were obtained using a Perkin Elmer spectrophotometer (L16000300 Spectrum Two LiTa, Llantrisant, United Kingdom) in accordance with the potassium bromide (KBr) pellet method. The samples were measured at wavelengths from 500 to 4000 cm^−1^ (Merga et al. 2007; Iravani et al. 2014).

## 6 Antibacterial Effect Test

The experiments on antimicrobial activity were carried out following the guidelines established by the Clinical and Laboratory Standards Institute (CLSI) (Hindler and Stelling 2007). The bacterial strain *E. faecalis* ATCC 29212 was used, and the turbidity of the suspension was adjusted to match the 0.5 McFarland standard (equivalent to 1.5 × 10^8^ CFU/mL). These standardized suspensions must be used within 15 min of preparation.

For the direct colony suspension method, the colonies should not exceed 18–24 h of isolation. The inoculum was standardized at the same time the suspension was prepared by placing the colonies in saline solution or broth (Mueller‐Hinton). The inoculum was then adjusted to a turbidity equivalent to the 0.5 McFarland standard.

Subsequently, a sterile cotton swab was immersed in the suspension, pressing the excess liquid against the wall of the tube. The entire plate was inoculated with the swab, starting from the top and streaking back and forth from one edge to the other. The plate was then rotated approximately 60°, and the streaking procedure was repeated, followed by a third rotation and streaking to ensure that the inoculum was evenly distributed. Three wells were created in the agar using a punch, where the following were added: Group 1, Vitapex; Group 2, AgNPs; and Group 3, Vitapex with AgNPs. The plates were then incubated, and after 24 h, the antibacterial effects of the study groups were observed. All tests were performed in triplicate (Humphries et al. 2021; Unver and Erenler 2020). After 24 h of incubation at 37°C, inhibition zone diameters were determined in triplicate using ImageJ software. Following scale calibration, measurements were obtained in millimeters via the circular selection tool and subsequently averaged to evaluate microbial susceptibility.

## 7 Results

**Figure 1:**
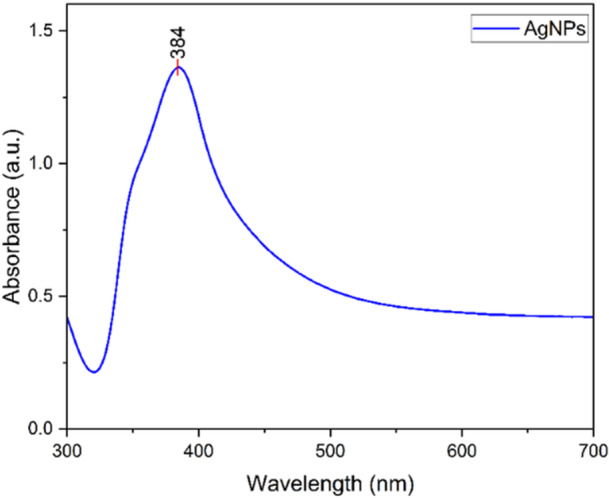
UV‐Vis spectrum obtained from AgNPs synthesized by the chemical method.

The characteristic surface plasmon resonance band of the AgNPs was observed at 384 nm using UV‐Vis spectroscopy (Figure 1). The narrow bandwidth suggested spherical AgNPs with low polydispersity, as confirmed by the TEM micrographs.

**Figure 2:**
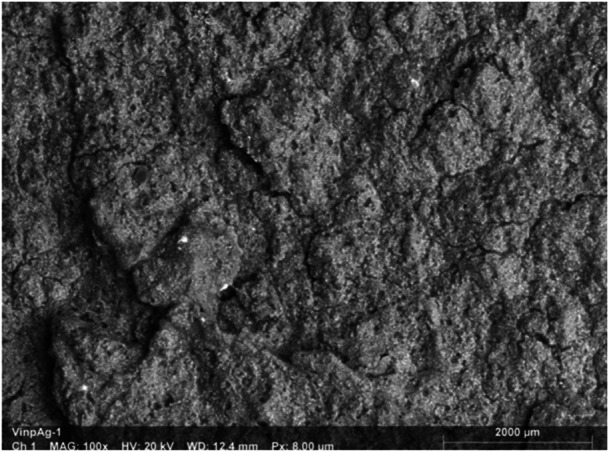
Representative micrograph of the AgNPs incorporated into Vitapex, obtained by SEM at 100× magnification.

Scanning electron microscopy (SEM) was performed to determine the homogeneity of the AgNPs incorporated into Vitapex (Figure 2). The images revealed a uniform surface morphology of the filling material.

**Figure 3:**
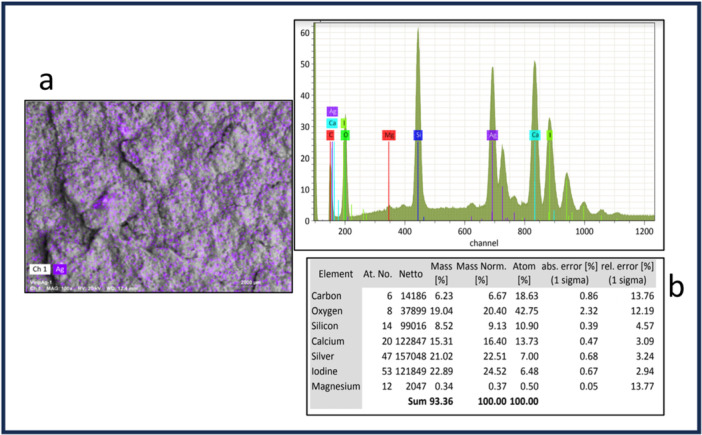
SEM‐EDS analysis: (a) Elemental chemical mapping showing the distribution of Ag in the sample; (b) percentage of Ag in the sample.

Elemental chemical mapping illustrated the distribution of the AgNPs across the entire surface of the sample (Figure 3a), as well as percentages of the chemical elements that made up the sample, with silver showing a weight percentage of 22.51% (Figure 3b).

**Figure 4:**
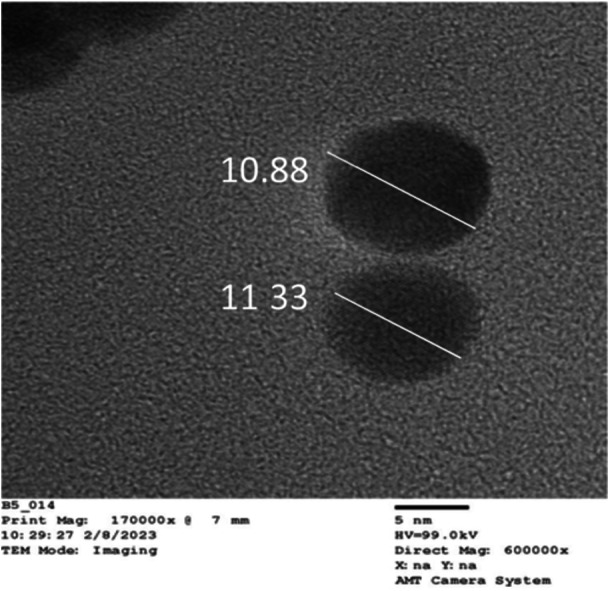
TEM micrograph of AgNPs.

As expected from the UV‐Vis results, the AgNPs exhibited a spherical morphology (Figure 4). They displayed uniform size distribution and were smaller than 50 nm, which is advantageous for the intended application.

**Figure 5:**
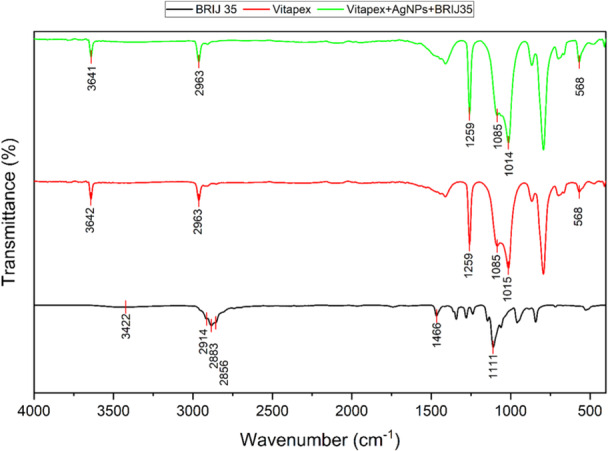
FTIR spectrum of the incorporation of Vitapex with the surfactant and AgNPs.

Subsequently, the iodoform paste was analyzed using infrared spectroscopy to identify the functional groups in Vitapex. Peaks were observed at 1599, 1496, and 1261 cm^−1^, which, according to the literature, correspond to the C–OH, Ca(OH)_2_, and N–NO_2_ groups, respectively—matching the infrared spectra of calcium hydroxide, iodoform, and silicone oil. These results are consistent with the infrared spectrum of Vitapex (Figure 5).

## 8 Antibacterial Effect

**Figure 6:**
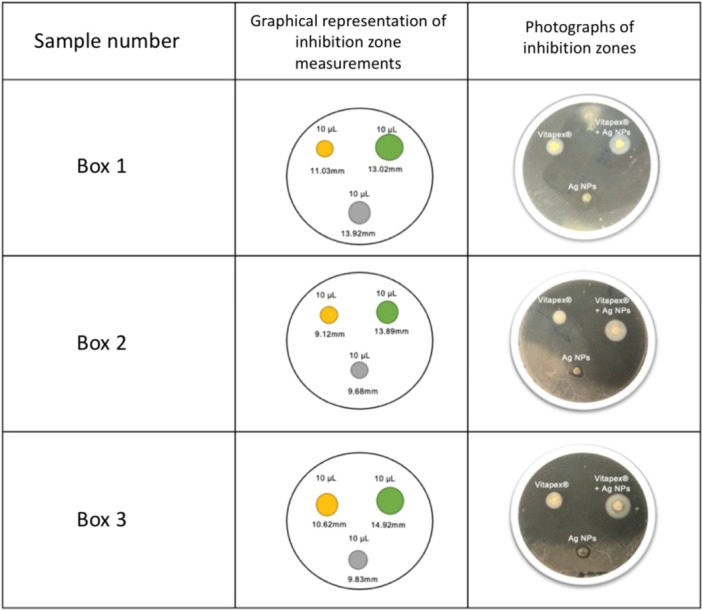
Representation of the distribution of experimental groups and their inhibition zones.

Once the *E. faecalis* ATCC 29212 strain was inoculated and bacterial growth achieved, the antibacterial effects of Vitapex, Vitapex with AgNPs, and AgNPs alone were confirmed. All showed an inhibitory effect against *E. faecalis* (Figure 6).

The following measurements were obtained for the inhibition zones of the three experimental groups: a mean of 10.25, with a standard deviation (SD) of 0.88, a minimum of 9.07, and a maximum of 11.11 for the Vitapex group; a mean of 13.92, with an SD of 0.82, a minimum of 12.95, and a maximum of 15.15 for the Vitapex with AgNPs group; and a mean of 11.14, with an SD of 2.39, a minimum of 9.28, and a maximum of 15.88 for the AgNP group (Table 1).

**Table 1:** Descriptive statistics and comparative analysis of the inhibition zones produced by the three experimental groups against *E. faecalis*.

Groups	Mean ± SD	Median	95% Confidence interval	*p* *
Vitapex	10.26 ± 0.88	10.72	9.58–10.94	0.002
Vitapex+AgNPs	13.93 ± 0.83	13.76	13.29–14.57	
AgNPs	11.15 ± 2.40	10.35	9.31–12.99	

*Note:* Kruskal Wallis.

*p ≤ 0.05.

**Figure 7:**
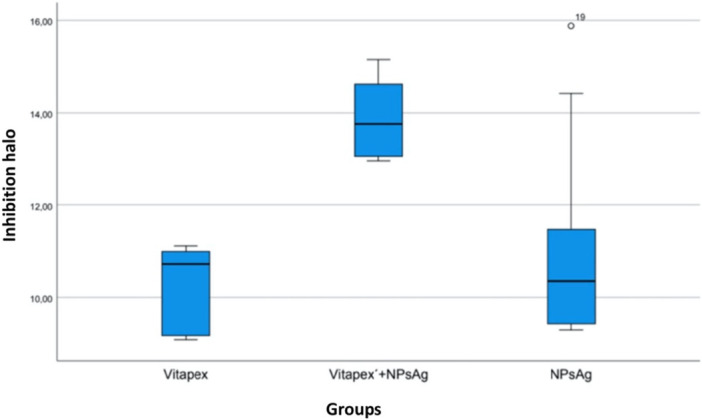
Bonferroni pairwise comparison between study groups.

Pairwise comparisons using the Bonferroni correction showed a significant difference between the Vitapex and Vitapex+AgNPs groups (*p* = 0.003), and between the AgNPs and Vitapex+AgNPs groups (*p* = 0.003), but not between the Vitapex and AgNPs groups (Figure 7).

## 9 Discussion

Complete removal of biofilm in primary and secondary infections of deciduous teeth cannot be achieved due to the complex and highly variable morphology of the roots and their canals (Afkhami et al. 2015). Irrigants and intracanal medications should remain in contact for as long as possible to enhance their disinfection efficacy (Wong et al. 2021). In addition, complementary disinfection strategies are necessary to improve the elimination of microorganisms in the root canals, especially *E. faecalis*, which is among the most persistent even after root canal treatment (Zou et al. 2024).

Vitapex, the most commonly used intracanal material for obturating root canals in primary teeth, possesses antibacterial properties in addition to being biocompatible with the periradicular tissues; it is therefore unlikely to harm the successor tooth. Furthermore, it is easy to handle and resorbs at a rate similar to that of primary tooth roots (Kratunova and Silva 2018). The combination of materials that enhance the antibacterial effect inside the root canal once it is filled will enable more successful treatments and optimal clinical conditions for patients.

Shrestha et al. reported that the effectiveness of silver nanoparticles (AgNPs) depends on the concentration and interaction time (Shrestha and Kishen 2016). Incorporating AgNPs into iodoform paste thus enhances its antibacterial effect owing to its sustained presence in the filled canal.

Afkhami et al. (2015) and Paula et al. (2015) demonstrated that Vitapex exerts only a mild antibacterial effect—particularly against *E. faecalis—*when compared with chlorhexidine and zinc oxide eugenol in vitro. To date, no studies have examined the incorporation of AgNPs into iodoform‐based pastes such as Vitapex, which contains 30.3% calcium hydroxide; however, several investigations have evaluated the addition of AgNPs to calcium hydroxide. For instance, Balto et al. (2020) incorporated 0.02% AgNPs into calcium hydroxide and observed a significant antibacterial effect against *E. faecalis* biofilms, attributed to the prolonged contact of the intracanal medication. Similarly, Rao et al. (2025) reported favorable results, with a marked reduction in biofilm formation after adding AgNPs to calcium hydroxide.

Consistent with Balto's et al. (2020) research, the present study combined iodoform‐based paste (Vitapex) with 0.02% AgNPs, producing a significantly larger inhibition zone than either AgNPs or Vitapex alone.

A key limitation of this study was its cross‐sectional design. Future research should evaluate the antibacterial effect at multiple time points—72 h, 1 week, and 1 month—to determine its persistence over the medium and long term.

## 10 Conclusion

Our results demonstrate that both Vitapex and AgNPs exhibit antibacterial activity against *E. faecalis*. When compared individually, no statistically significant difference is observed in their efficacy; however, their use in combination demonstrates a synergistic effect resulting in increased inhibition, particularly in short‐term treatments. This enhanced activity represents a promising strategy for endodontic therapies, optimizing the elimination of the pathogen.

## Author Contributions

Carbajal‐Bastida Sarasuadi, Morales‐Luckie Raúl Alberto, and María Guadalupe González‐Pedroza implemented the experimental process. Guadarrama‐Reyes Saraí Carmina and Bermeo‐Escalona Josué Roberto performed the statistical analysis and description. Carbajal‐Bastida Sarasuadi wrote the manuscript. Robles‐Bermeo Norma Leticia critically reviewed the manuscript and direction of the project. Carlo Eduardo Medina Solís performed the final revision of the manuscript.

## Funding

The authors have nothing to report.

## Conflicts of Interest

The authors declare no conflicts of interest.

## Data Availability

All data generated or analyzed during this study are included in this published manuscript.
